# Parental Preferences of Influenza Vaccination for Children in China: A National Survey with a Discrete Choice Experiment

**DOI:** 10.3390/ijerph19042145

**Published:** 2022-02-14

**Authors:** Minghuan Jiang, Yilin Gong, Yu Fang, Xuelin Yao, Liuxin Feng, Shan Zhu, Jin Peng, Xinke Shi

**Affiliations:** 1 Department of Pharmacy Administration and Clinical Pharmacy, School of Pharmacy, Xi’an Jiaotong University, Xi’an 710061, China; yilingong0601@stu.xjtu.edu.cn (Y.G.); yufang@mail.xjtu.edu.cn (Y.F.); 15291832700@163.com (X.Y.); zoey0323@163.com (S.Z.); pengjin143@163.com (J.P.); 2 Center for Drug Safety and Policy Research, Xi’an Jiaotong University, Xi’an 710061, China; 3 Shaanxi Center for Health Reform and Development Research, Xi’an 710061, China; 4 Research Institute for Drug Safety and Monitoring, Institute of Pharmaceutical Science and Technology, Western China Science & Technology Innovation Harbor, Xi’an 712000, China; 5 Department of Pharmacy, The Second Affiliated Hospital of Xi’an Jiaotong University, Xi’an 710004, China; jdff369@163.com; 6 Health Science Center, Xi’an Jiaotong University, Xi’an 710061, China; 309819@stu.xjtu.edu.cn

**Keywords:** influenza vaccine, discrete choice experiment, children, preference, willingness to pay, vaccine uptake

## Abstract

The influenza vaccination coverage among children is low in China. We aimed to conduct a nationwide survey to quantify parental preferences and willingness to pay (WTP) for influenza vaccination for their children. Parents with children aged six months to 18 years from six provinces in China were investigated by a discrete choice experiment regarding six influenza vaccination attributes. Mixed logit models were used to estimate the relative importance of vaccine attributes and parents’ WTP. Interaction analysis and subgroup analysis were conducted to explore preference heterogeneity. A total of 1206 parents were included in the analysis. Parents reported vaccine effectiveness as the most important vaccine attribute. The mode of vaccine administration had no significant impact on parents’ preferences. Parents aged over 30 years with higher education or income levels were more likely to prefer no influenza vaccination for their children. The largest marginal WTP (CNY 802.57) for vaccination and the largest increase in vaccine uptake (41.85%) occurred with improved vaccine effectiveness from 30% to 80%. Parents from central regions or mid-latitude areas had a relatively lower WTP than those from other regions. No significant difference in the relative importance of vaccine attributes were observed among parents from various regions of China.

## 1. Introduction

The incidence of influenza infection and its complications are highest among children, reaching 50% in epidemic seasons [1]. A global survey showed that the influenza virus causes 10.1 million cases of acute lower respiratory infection and 15,300 in-hospital deaths among children under five years of age annually [2]. In China, the number of influenza-related outpatient consultations for children under 15 years old is 4.1-fold greater than the number for adults aged 60 years or above [3]. Additionally, children play an important role in the spread of influenza in schools, households, and communities, causing a large number of school-age children to be absent from school and parents to be absent from work, which results in large disease and economic burdens [4,5].

Influenza vaccination is the most effective way to protect against influenza infection [6,7,8]. Previous studies support the cost-effectiveness of influenza vaccination among children in Europe [9], the United States [10], Canada [11], and Hong Kong [12]. Over 40% of countries or regions worldwide have implemented free influenza vaccination programs [13], except China. The technical guidelines for seasonal influenza vaccination in China state that children aged six months to five years are a priority population for influenza vaccination [1]. However, the current coverage of influenza vaccination among Chinese children is relatively low (11%) [14], far below the rate (60–70%) in developed countries such as European countries and the United States [13]. In China, it was reported that 76.09% of people learned more knowledge of influenza and 80.9% of parents planned to have their children receive the influenza vaccination after the COVID-19 pandemic, which could potentially increase the actual coverage of influenza vaccination [15,16].

Parental preferences toward vaccination are pivotal for policy makers to tailor immunization programs and improve the uptake of vaccines for children [17]. A discrete choice experiment (DCE) is the most widely used quantitative method to explore individuals’ preferences in the field of health economics and policy research including vaccination programs [18,19,20]. Although several prior studies on parental preferences regarding influenza vaccination have been conducted in developed countries [6,8], findings on the most influential attributes of influenza vaccination are inconsistent, and the results from other countries might not directly apply to China due to cultural differences [21]. To the best of our knowledge, no studies specifically investigating parental preferences for influenza vaccination have been conducted in China. Therefore, we aimed to design a nationwide DCE study to quantify the parents’ preferences and their willingness to pay (WTP) for influenza vaccination for children and to further explore optimal strategies to expand the vaccination coverage in China. 

## 2. Materials and Methods

### 2.1. Attributes and Levels 

In the application of DCE studies for vaccination programs, the individuals’ preferences toward vaccination are assumed to rely on vaccine attributes and attribute levels. In the present study, we retrieved initial information on attributes and levels from similar published DCE studies on influenza vaccination [6,7,8,17,22,23,24]. To determine the final attributes in our survey, we conducted face-to-face semi-structured interviews with 10 doctors from three community health care centers in Xi’an, Shaanxi Province. The doctors included five pediatricians with at least 10-years of working experience and five vaccination clinicians with at least 5-year vaccination experience. Thereafter, we designed a pre-survey questionnaire and conducted a pilot study among 30 randomly selected parents of children aged six months to 18 years from the three community health care centers as above-mentioned in Xi’an. We finally identified six attributes in the present study including vaccine effectiveness, vaccine safety (probability of mild side effects), source of recommendation for vaccination, vaccination cost, duration of vaccination protection, and mode of administration. 

### 2.2. DCE Design and Questionnaire 

The corresponding levels of each attribute are shown in Table 1. Participants made trade-offs between various levels of attributes (one attribute with four levels, three attributes with three levels, and two attributes with two levels), and selected their preferred vaccination strategy. If using full-factorial design, 432 hypothetical influenza vaccination alternatives and 93,096 choice tasks were generated. It is not feasible to present all choice tasks to one individual in a survey. Therefore, we performed fractional factorial design using orthogonal arrays [21] to select 72 vaccination alternatives from the pool, with R version 4.0.2 (The R Project for Statistical Computing, Vienna, Austria). In line with the principles of orthogonality, level balance, and minimum overlap [25], 36 choice sets were produced.

In a pilot test, we separated the 36 choice sets into three versions of the DCE questionnaire, with each version comprising 12 choice tasks. However, most participants had difficulty completing the test due to its complexity. For the formal survey, we further divided the 36 choice sets into six versions with six choice sets in each version. To examine the validity of the survey, we added another two choice sets in a test of rationality; participants who failed the test were considered “irrational” or not having a good understanding of the DCE design. The data for those who failed in the rational test were excluded from the final analysis. The formal questionnaire in our survey included participants’ sociodemographic information and eight choice sets in the DCE design. Each choice set comprised three options, with two vaccination options and one opt-out option for no vaccination [17], as shown in Supplementary Table S1. Participants were asked to choose one of the three options in each choice set. 

### 2.3. Participants and Data Collection

We conducted the formal survey using the largest online platform for questionnaire surveys (Wen Juan Xing) in China [26] where there are 2.6 million registered members on the platform. We selected six provinces (Guangdong, Liaoning, Hubei, Jiangxi, Shaanxi, Yunnan) for sampling on the basis of the latitude of the capital city in each province and its geographic location and economic level, as shown in Supplementary Table S2 and Figure S1. 

We conducted the online survey from January to February 2021. To ensure no survey stress for the participants and improve the validity of this study, the survey was anonymous. The platform identified the respondents’ provincial information to guarantee the inclusion of participants from corresponding provinces. Eligible participants had to have children aged between six months to 18 years. If respondents had more than two children, the information collected in the survey referred to their youngest child. Prior to the formal survey, all participants received a questionnaire link to an authenticity test, which included the following three questions: (1) How old are you?; (2) What is your marital status?; and (3) What are the ages of your children? Participants who answered that they were aged less than 20 years, not married, or had children outside of the age range six months to 18 years were considered ineligible and could not continue with the formal survey. The questionnaire link was invalid once the authenticity test was completed.

The minimum sample size was 500, calculated using the rule of thumb (Equation (1)) proposed by Orme [27].

(1) n=500c/(t*a)

where, *c* represents the number of analysis cells. Because we considered two-way interactions between sociodemographic characteristics and vaccine attributes, *c* is equal to the largest product of levels [28] of any two attributes (*c* = 4*3), *t* is the number of choice tasks (*t* = 6), and *a* is the number of alternatives in each choice set (*a* = 2, excluding the opt-out option [21]). 

### 2.4. Data Analysis

Data analyses were conducted using Stata version 15.0 (StataCorp LLC, College Station, TX, USA). The results of descriptive analysis regarding the participants’ sociodemographic characteristics are presented as number and percentage. A mixed logit model was used to estimate the respondents’ preferences for different vaccine attributes, with main effects and two-way interaction effects for preference heterogeneity [29]. Participants chose one option from each choice task with the largest utility; the random utility model was applied using Equation (2)

*U_n_* = *V_n_* + *ε_n_* = *β*_0_ + *β*_1_ × Effectiveness_50%_ + *β*_2_ × Effectiveness_80%_
+ *β*_3_ × Recommendation_doctor_ + *β*_4_ × Recommendation_school_
+ *β*_5_ × Protection duration_12-month_ + *β*_6_ × Safety_0%_ + *β*_7_ × Safety_15%_
+ *β*_8_ × Way_nasal spray_ + *β*_9_ × Cost + *ε_n_* (2)

where, *U_n_* is the utility for an alternative to influenza vaccination and *V_n_* is the deterministic utility of six observed vaccine attributes. *ε_n_* is the random error term. [25]. *β*_0_ is a random alternative specific constant (ASC) [30] that represents the preference for vaccination alternatives compared with no vaccination (opt-out option). *β*_1_ to *β*_8_ values provide quantitative information on the relative importance (preference weights) for each attribute level compared with the reference level. The sign of the coefficients reflects the direction of the positive or negative effect on utility. We set the vaccine price as a continuous variable and other attributes as categorical variables. To quantify the importance of different attributes, we evaluated the relative importance score of the six attributes [31]. We first calculated the difference in coefficient values between the highest and lowest level for a single attribute, then divided this by the sum of the differences of all attributes included in the study; the importance of the attribute is positively correlated with the score. 

We also explored the interactive effects of the participants’ sociodemographic characteristics, no vaccination (denoted Neither), and different attribute levels on parents’ preferences in the mixed logit model. Due to the difference socioeconomic levels and peak times of influenza in different latitude regions, we further assessed the heterogeneity of parental preferences in diverse regions of China. In the present study, we performed subgroup analyses based on different geographic regions and latitudes of the six sampled provinces. The six provinces were divided into eastern, western, and central regions of China, and high, middle, and low latitudes, according to the Technical Guidelines for Seasonal Influenza Vaccination in China [1]. 

### 2.5. Policy Analysis 

Participants’ WTP for a change in attribute levels was calculated using the ratio attained by dividing the coefficients of the categorical variables by the coefficient of the vaccination cost. For instance, assuming that other attribute levels were equal, *β*_1_/*β*_9_ represents the parents’ WTP for increasing vaccine effectiveness from 30% to 50%. Additionally, we predicated the uptake rates of influenza vaccination under various scenarios using Equation (3) [32]:

(3) $$P_{i}=\frac{e^{U_{i}}}{\sum e^{U_{j}}}$$

where, *P_i_* is the probability of choosing alternative *i* among a set of *j* alternatives; *U_i_* is the total utility for the vaccination program; *U_j_* is the utility of both vaccination and no vaccination. We set the base-case vaccination program with the following attribute levels: CNY 150 as the vaccination cost, vaccination recommended by relatives, 30% vaccine effectiveness, 30% probability of mild side effects for vaccine safety, intramuscular injection for mode of vaccination, and 6-month protection duration of vaccination. The uptake rate in the base-case scenario was determined, and the change in vaccination uptake was assessed using a one-way variation in specific attributes.

## 3. Results

### 3.1. Participants’ Sociodemographic Information

The sociodemographic characteristics of all respondents are shown in Table 2. A total of 1534 parents completed the formal survey, 1206 (78.62%) passed the validity test. Among the 328 exclusive respondents, 126 and 202 failed in one and two choice sets for the rational tests, respectively. We carefully reviewed the respondents’ answers and excluded them one by one. Among all respondents who passed the validity test, 667 were women (55.31%) and 80.84% had a high education level (bachelor/college or above); 61.85% of respondents were aged 31 to 40 years. A total of 656 (54.39%) respondents’ children were boys, and 66.67% of children were aged below seven years. There were 394 respondents from the eastern region (Guangdong and Liaoning), 403 from the central region (Hubei and Jiangxi), and 409 from the western region (Shaanxi and Yunnan). A total of 1022 parents (84.74%) reported that their children were in good health and 432 (35.82%) felt that the probability of their children developing influenza infection was low.

### 3.2. Parental Preferences toward Influenza Vaccination

The results of the mixed logit model on the main effects (model 1) and main effects with two-way interaction effects (model 2) are shown in Table 3. In model 1, the coefficients of all attribute levels were statistically significant (*p* < 0.01), except for the mode of administration. The positive coefficients indicated that parents preferred influenza vaccination with higher effectiveness, lower risk of mild side effects, vaccination recommended by a school or physician, and longer duration of protection. The negative coefficient for vaccination cost suggested that parents preferred vaccination with a lower cost. The positive sign of the ASC coefficient indicated that parents preferred to vaccinate their children to prevent influenza infection. 

In model 1, there were several coefficients with a significant standard deviation, indicating that heterogeneity existed in the parents’ preferences toward vaccination. In model 2, we explored the preference heterogeneity with interaction terms between sociodemographic characteristics and attribute levels. The positive estimators were correlated with greater preference for no vaccination (Neither) and attribute levels. For the “Neither” interaction terms, parents aged over 30 years and those with a college degree or above or high income (annual household income more than CNY 100,000) preferred “no vaccination” over vaccination. For other interaction terms, parents with high levels of education (master’s degree or above) or high incomes preferred vaccination with a lower risk of mild side effects. Urban residents preferred vaccines with higher effectiveness and longer protection duration. Participants who had boys preferred vaccination via nasal spray.

**Figure 1:**
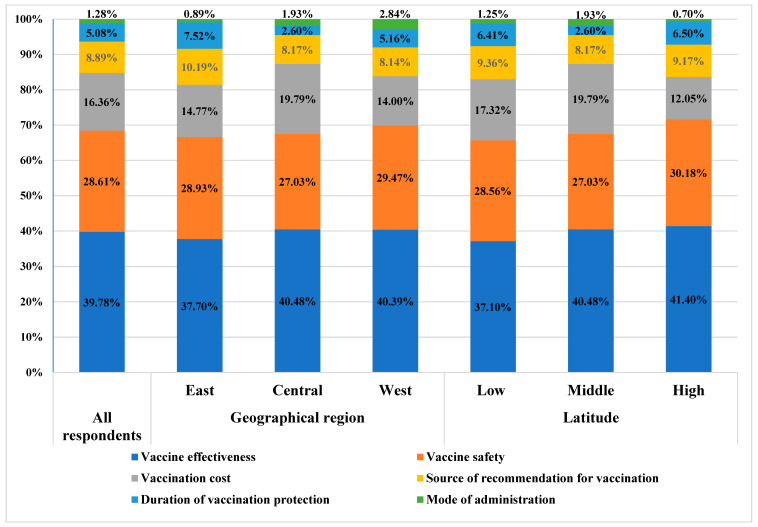
Relative importance of six attributes in all respondents and subgroups.

The relative importance of different vaccine attributes is shown in Figure 1. Vaccine effectiveness was the most important attribute, with the highest score (39.78%), and mode of administration was the least important attribute (1.28%). The results of the subgroup analyses are shown in Figure 1 and Supplementary Table S3. The findings in the main effects models and rankings of the relative importance of vaccine attributes were consistent with the findings for all respondents.

### 3.3. Policy Analysis 

#### 3.3.1. Trade-Offs among Attributes

The results regarding WTP for specific attribute levels are shown in Table 4. For all respondents, parents were willing to pay an additional CNY 268.83 and CNY 802.57 if the vaccine effectiveness was increased from 30% to 50% and 80%, respectively. The WTP for mild vaccination side effects decreasing from 30% to 0% and 15% was CNY 557.29 and CNY 224.18, respectively. Parents were willing to pay an extra CNY 102.57 to receive an influenza vaccination with 12-month protection compared with a short duration of six months. Compared with a recommendation for vaccination from relatives, parents preferred a recommendation from physicians and schools at an additional cost of CNY 179.46 and CNY 82.97, respectively.

Parents from western China had relative higher WTP for vaccine effectiveness (CNY 952.15 for 80% effectiveness) and vaccine safety (CNY 694.71 for 0% mild side effects) compared with parents from eastern and central regions. Parents in the eastern region had relatively higher WTP for vaccination recommended by a physician (CNY 227.69) and 12-month vaccination protection (CNY 168.14). Parents from low-latitude areas had relatively higher WTP for vaccine effectiveness (CNY 1134.08 for 80% effectiveness), vaccine safety (CNY 826.80 for 0% mild side effects), vaccination recommended by a physician (CNY 251.33), and 12-month vaccination protection (CNY 178.14) compared with parents from mid- and high-latitude regions. 

#### 3.3.2. Probability of Vaccine Uptake

**Figure 2:**
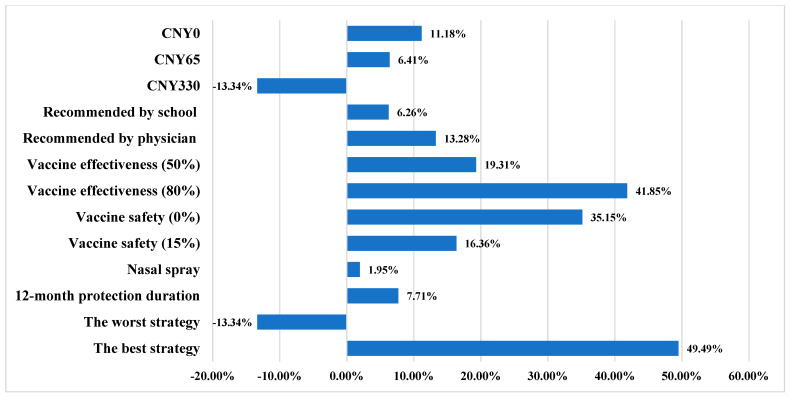
One-way variations in probability of vaccine uptake. CNY: Chinese Yuan.

The uptake rate in the base-case scenario was estimated to be 50.14%. The change in vaccine uptake with one-way variation in vaccine attribute levels is shown in Figure 2. The uptake of influenza vaccination would increase 41.85% by increasing vaccine effectiveness from 30% to 80%. When vaccine safety decreased from 30% to 0%, vaccine uptake increased 35.15%. Implementation of a free vaccination program would result in an 11.18% increase in the vaccination rate.

Under the scenario of the best vaccination strategy (80% vaccine effectiveness, physician recommendation for vaccination, 12-month duration of protection, no mild side effects, intramuscular injection, free vaccination), the uptake rate would increase 49.49%. In contrast, uptake would decrease 13.34% under the worst vaccination scenario (30% vaccine effectiveness, vaccination recommended by relatives, 6-month vaccination protection, 30% mild side effects, intramuscular injection, and CNY 330 vaccination cost).

## 4. Discussion

Our study showed that parents in China preferred influenza vaccination with a high vaccine effectiveness, low risk of mild side effects, vaccination recommendation from a physician, long duration of protection, and low cost of vaccination. Findings on the relative importance of vaccine attributes were consistent among parents from different regions of China. Parents from eastern and western regions (or high/low latitude areas) were willing to pay more for additional benefits gained through better vaccine attributes compared with parents from the central regions (or mid-latitude areas). 

In the present study, we found that influenza vaccine effectiveness was the most important attribute for parents regarding vaccinating their children. Two previous studies in the United States support the findings that vaccine effectiveness is the most important attribute among both children and their parents [7,8]. A systematic review on individual preferences toward vaccine attributes among children and adolescents showed that vaccine effectiveness and the risk of vaccination side effects were the most widely reported attributes in DCE studies [33]; these were also found to be the most important vaccine attributes for decision makers [17,26,29,32]. However, the risk of severe side effects was the most important factor in one study from China assessing the preferences of 428 parents in vaccinating their children (no specific vaccine types) [29]; this result was likely due to the different definition of vaccine safety in that study versus the mild side effects defined in our study.

DCE studies are widely used in vaccine policy analysis to predict the probability of vaccine uptake with different combinations of vaccine attributes [25], which provides evidence for policy makers to make decisions regarding immunization programs [6,17,21,22]. In China, the effectiveness of influenza vaccination varies according to season and is estimated to be 18–57%, with the highest rate among children aged 6–35 months who receive two doses [34]. In our study, we predicted that the influenza vaccine uptake rate would increase 19.31–41.85% when vaccine effectiveness improved from 30% to 50–80%. The uptake rate would increase 16.36–35.35% if mild vaccination side effects decreased from 30% to 0–15%. This suggests that the current impact of influenza vaccination is moderate, and better effectiveness and safety against confirmed influenza infection is needed to further improve belief in the benefits of influenza vaccination among parents. 

The cost of vaccination was the third most important attribute in our study population, and the vaccination rate increased with decreased vaccination cost [35]. The coverage of influenza vaccination greatly improves after the implementation of a free vaccination policy, especially in developed countries such as Brazil [36], Australia [37], and Korea [38]. In China, Beijing has implemented a free influenza vaccination program for primary and middle school students (in 2007). The vaccination rate reached 40–70% during the period 2007–2016 [39], which is nearly four-fold the rate before 2007 [40]. To further improve the coverage of influenza vaccination among children in China, it is necessary to consider accelerating national or regional immunization programs for influenza vaccination among children. The cost-effectiveness of such programs should be further examined to support its implementation. 

Since a live attenuated influenza vaccine became available in 2020 in China, no studies have explored parental preferences regarding the mode of vaccine administration (nasal spray versus intramuscular injection). Previous studies have shown that the preference for the live attenuated influenza vaccine is due to the children’s fear of needles, and the preference for inactivated influenza vaccine is due to the belief that intramuscular injection is more effective [41]. In the present study, we found that parents had no significant preference between the two modes of vaccination. These findings were consistent with results reported in studies from the United States [8] and Japan [6], reporting that parents’ preferences for vaccination were not associated with the mode of vaccine administration. However, the results of another study from the United States that investigated children’s preferences regarding influenza vaccination showed that 74% of children preferred the nasal spray over injection mainly because of pain at the time of and following injection [7].

There were several limitations in our study. First, we performed an online survey because it was difficult to conduct an onsite questionnaire survey during the COVID-19 pandemic. In China, most parents with children aged six months to 18 years are able to access the Internet and have no difficulty in completing surveys. We performed tests of authenticity and rationality to examine the validity of our survey. Second, most registered members on the survey platform were from urban areas, which cannot fully represent the preferences of parents from rural areas. Our study found that parents with higher levels of education or incomes preferred vaccination with a lower risk of mild side effects, which indicated that the relative importance of vaccine safety may decrease if more rural parents are included in the study. Rural parents may possibly have greater preferences toward cost or source of recommendation for vaccination. Further studies among broader general populations are warranted to examine the robustness of our findings. Third, the vaccination coverage of respondent parents in our study was unsure, which may generate interaction with parents’ preferences toward different attributes. Finally, the COVID-19 pandemic improved the individuals’ willingness to receive influenza vaccination [42,43]; however, its impact could not be explored in the present study. 

## 5. Conclusions

Our findings suggest that influenza vaccine effectiveness was the parents’ most preferred attribute with respect to vaccinating their children, and they had no specific preference difference regarding the mode of vaccine administration. Parental preferences regarding the importance of vaccine attributes did not differ across regions whereas parents from central regions or mid-latitude areas of China had relatively lower WTP for influenza vaccination. Policy makers can implement effective interventions such as promoting vaccination recommendations from physicians or considering the inclusion of influenza vaccination in immunization programs to improve the uptake rate of influenza vaccination in China.

## Figures and Tables

**Table 1:** Attributes and levels included in the DCE design.

Attributes	Levels of Attributes
Cost of vaccination (CNY)	0, 65, 150, 330
Source of recommendation for vaccination	School, physician, relatives
Vaccine effectiveness (%)	30, 50, 80
Vaccine safety * (%)	0, 15, 30
Mode of administration	Nasal spray, intramuscular injection
Duration of vaccination protection (months)	6, 12

CNY: Chinese Yuan (1 USD equals to CNY 6.89 in 2020); Vaccine safety *: the probability of mild side effects.

**Table 2:** Socio-demographic characteristics of all respondents (N = 1206).

Characteristics	N	Proportion (%)
Gender of parents		
Male	539	44.69
Female	667	55.31
Age of parents (years)		
≤30	284	23.55
31–40	746	61.85
≥41	176	14.60
Marital status		
Married	1193	98.92
Divorced or widowed	13	1.08
Education level		
Master or above	108	8.96
Bachelor or college	975	80.84
High school (secondary school) and below	123	10.20
Province for living		
Guangdong	199	16.50
Liaoning	195	16.17
Hubei	200	16.58
Jiangxi	203	16.83
Shaanxi	216	17.91
Yunnan	193	16.01
Living residence		
Urban	1123	93.12
Rural	83	6.88
Healthcare-related work		
Yes	108	8.96
No	1098	91.04
Family annual income (CNY)		
≤100,000	310	25.71
100,000–200,000	648	53.73
≥200,000	248	20.56
Age of the youngest children		
6 months–3 years	389	32.26
3–7 years	415	34.41
7–12 years	265	21.97
12–17 years	137	11.36
Gender of children		
Boy	656	54.39
Girl	550	45.61
Health condition of children		
Good	1022	84.74
Average	169	14.01
Poor	15	1.25
Probability of children infected by flu annually		
Low	432	35.82
Average	481	39.88
High	293	24.30

CNY: Chinese Yuan (1 USD equals to CNY 6.89 in 2020).

**Table 3:** Results of mixed logit model with main effects and interactions in all respondents.

Attributes (Ref.)	Model 1				Model 2	
	Coeff. (*β*)	SE^+^	SD	SE^++^	Coeff. (*β*)	SE^+^
Vaccination cost	−0.003 ^###^	<0.001			−0.003 ^###^	<0.001
Vaccine effectiveness (30%)						
50%	0.816 ^###^	0.060	0.421 ^##^	0.163	0.463 ^##^	0.191
80%	2.436 ^###^	0.110	1.738 ^###^	0.109	1.563 ^###^	0.289
Source of recommendation (relatives)						
Physician	0.545 ^###^	0.059	0.621 ^###^	0.113	0.551 ^###^	0.059
School	0.252 ^###^	0.056	0.025	0.247	0.252 ^###^	0.056
Duration of vaccination protection (6 months)						
12 months	0.311 ^###^	0.051	0.441 ^###^	0.147	−0.094	0.174
Vaccine safety (30%)						
0%	1.752 ^###^	0.089	1.129 ^###^	0.096	1.016 ^###^	0.203
15%	0.680 ^###^	0.060	0.008	0.232	0.274 ^#^	0.160
Mode of administration (intramuscular injection)						
Nasal spray	0.078	0.052	0.448 ^###^	0.134	−0.022	0.073
ASC	0.461 ^###^	0.163	2.956 ^###^	0.156	2.832 ^###^	0.493
**Interaction terms**						
Neither * parents aged 30–40 years					0.723 ^###^	0.275
Neither * parents aged over 40 years					1.094 ^###^	0.380
Neither * master or above					1.763 ^###^	0.595
Neither * bachelor or college					1.069 ^##^	0.456
Neither * income ^a^					0.931 ^###^	0.315
Neither * income ^b^					1.460 ^###^	0.377
Vaccine safety (0%) * master or above					0.717 ^##^	0.322
Vaccine safety (0%) * bachelor					0.351	0.224
Vaccine safety (15%) * master or above					0.731 ^###^	0.261
Vaccine safety (15%) * bachelor					0.233	0.179
Vaccine effectiveness (50%) * urban					0.299	0.200
Vaccine effectiveness (80%) * urban					0.639 ^##^	0.299
Duration of vaccination protection_12-month_ * urban					0.445 ^##^	0.182
Vaccine effectiveness (50%) * income ^a^					0.142	0.130
Vaccine effectiveness (50%) * income ^b^					−0.013	0.164
Vaccine effectiveness (80%) * income ^a^					0.456 ^##^	0.189
Vaccine effectiveness (80%) * income ^b^					0.295	0.236
Vaccine safety (0%) * income ^a^					0.528 ^###^	0.168
Vaccine safety (0%) * income ^b^					0.718 ^###^	0.211
Vaccine safety (15%) * income ^a^					0.232 ^#^	0.134
Vaccine safety (15%) * income ^b^					0.282 ^#^	0.169
Nasal spray * boy					0.200 ^##^	0.096
AIC	10,945.28				10,894.32	
BIC	11,097				11,229.71	
No. of respondents, (n)	1206				1206	
No. of observations, (n)	21,708				21,708	
Log-likelihood	−5453.6385				−5405.1589	
Likelihood ratio chi2	1357.07				1320.43	

^#^*p* < 0.1, ^##^ *p* < 0.05, ^###^ *p* < 0.01; ASC: Alternative specific constant; income ^a^: Annual household income is CNY 100,000–200,000 (15,650–31,300 USD); income ^b^: Annual household income over CNY 200,000 (31,300 USD); Neither: no vaccination; SD: standard deviation; SE: standard error; SE^+^: standard error of coefficient; SE^++^: standard error of standard deviation; AIC: Akaike Information Criterion; BIC: Bayesian Information Criterion; ASC: Alternative Specific Constant. * interaction terms.

**Table 4:** Parents’ willingness to pay for the attributes of influenza vaccination.

Attributes (Ref.)	All Respondents (N = 1206)	Geographical Region			Latitude		
		East (N = 394)	Central (N = 403)	West (N = 409)	High (N = 392)	Middle (N = 403)	Low (N = 411)
	WTP (95% CI)	WTP (95% CI)			WTP (95% CI)		
Vaccine effectiveness (30%)							
50%	268.83 (218.07, 319.59)	236.12 (147.36, 324.88)	265.91 (196.03, 335.79)	313.24 (196.44, 430.04)	215.24 (134.28, 296.20)	265.91 (196.03, 335.79)	339.11 (206.01, 472.22)
80%	802.57 (679.53, 925.61)	842.35 (601.79, 1082.92)	675.01 (527.40, 822.62)	952.15 (650.98, 1253.31)	707.17 (517.85, 896.50)	675.01 (527.40, 822.62)	1134.08 (747.27, 1520.89)
Source of recommendation (relatives)							
Physician	179.46 (135.46, 223.46)	227.69 (139.65, 315.72)	136.32 (77.71, 194.92)	192.01 (97.88, 286.14)	178.47 (103.95, 252.99)	136.32 (77.71, 194.92)	251.33 (137.10, 365.55)
School	82.97 (45.08, 120.86)	83.90 (12.72, 155.07)	62.04 (11.02, 113.05)	114.23 (30.42, 198.03)	103.76 (36.73, 170.80)	62.04 (11.02, 113.05)	98.04 (8.69, 187.40)
Duration of vaccination protection (6 months)							
12 months	102.57 (67.78, 137.35)	168.14 (95.21, 241.07)	43.33 (−0.93, 87.59)	121.71 (42.62, 200.80)	122.18 (58.76, 185.60)	43.33 (−0.93, 87.59)	178.14 (84.05, 272.22)
Vaccine safety (30%)							
0%	577.29 (485.79, 668.79)	646.32 (459.82, 832.82)	450.84 (346.54, 555.15)	694.71 (468.45, 920.97)	544.23 (392.48, 695.98)	450.84 (346.54, 555.15)	826.80 (543.02, 1110.58)
15%	224.18 (178.06, 270.29)	243.49 (154.12, 332.87)	178.36 (119.17, 237.56)	274.50 (167.01, 381.98)	217.14 (139.22, 295.06)	178.36 (119.17, 237.56)	312.64 (186.68, 438.61))
Way of vaccination (intramuscular)							
Nasal spray	25.81 (−9.00, 60.62)	−19.92 (−80.55, 40.71)	32.16 (−18.20, 82.52)	66.90 (−10.42, 144.22)	23.80 (−33.99, 81.58)	32.16 (−18.20, 82.52)	19.07 (−59.91, 98.04)

Eastern regions: Guangdong and Liaoning; Central regions: Hubei and Jiangxi; Western regions: Shaanxi and Yunnan; High latitude: Shaanxi and Liaoning; Middle latitude: Hubei and Jiangxi; Low latitude: Guangdong and Yunnan; WTP: Willingness to pay (Chinese Yuan).

## Data Availability

The data set used in the current study will be made available at a reasonable request to the corresponding author.

## Supplementary Materials

The following supporting information can be downloaded at: https://www.mdpi.com/article/10.3390/ijerph19042145/s1, Table S1: Illustration of choice sets in the DCE design, Table S2: Sampling of six provinces in China, Table S3: Results of mixed logit model with main effects in subgroup analyses, Figure S1: Locations of parents from six province in China.
