# Supplementary material for: Parental Preferences of Influenza Vaccination for Children in China: A National Survey with a Discrete Choice Experiment
Source: Int J Environ Res Public Health. 2022 Feb 14;19(4):2145. doi: 10.3390/ijerph19042145 (PMC8871809; doi:10.3390/ijerph19042145)

Supplementary

Table S1. Illustration of (a) choice sets and (b) rational test in the DCE design

(a)

Attributes	Strategy 1	Strategy 2	Strategy 3
Vaccine effectiveness	50%	30%	Opt-out
Cost of vaccination (CNY)	65	330	
Source of recommendation for vaccination	School	Physician	
Duration of vaccination protection (months)	6	12	
Vaccine safety	15%	30%	
Mode of administration	Nasal spray	Intramuscular injection	

Which strategy do you prefer: Strategy 1 ☐ Strategy 2 ☐ Strategy 3 ☐

(b)

Attributes	Strategy 1*	Strategy 2	Strategy 3
Vaccine effectiveness	80%	30%	Opt-out
Cost of vaccination (CNY)	0	0	
Source of recommendation for vaccination	Physician	Physician	
Duration of vaccination protection (months)	12	6	
Vaccine safety	0%	30%	
Mode of administration	Intramuscular injection	Intramuscular injection	

Which strategy do you prefer: Strategy 1 ☐ Strategy 2 ☐ Strategy 3 ☐

*Participants who failed to select strategy 1 were considered irrational in the survey.

Table S2. Sampling of six provinces in China

Province	Latitude*	Geographical region	Ranking of GDP per capita
Guangdong	Low	East	6
Yunnan	Low	West	24
Hubei	Middle	Central	8
Jiangxi	Middle	Central	21
Shaanxi	High	West	12
Liaoning	High	East	16

*The division of different latitudes was derived from the Technical guidelines for seasonal influenza vaccination in China (2020-2021).

Table S3. Results of mixed logit model with main effects in subgroup analyses

Attributes (Ref.)	Geographical region						Latitude					
	East (N=394))		Central (N=403)		West (N=409)		High (N=392)		Middle (N=403)		Low (N=411)	
	Coeff. (SE)	SD (SE)	Coeff. (SE)	SD (SE)	Coeff. (SE)	SD (SE)	Coeff. (SE)	SD (SE)	Coeff. (SE)	SD (SE)	Coeff. (SE)	SD (SE)
Vaccination cost	-0.003***	NA	-0.004***	NA	-0.002***	NA	-0.003***	NA	-0.004***	NA	-0.002***	NA
	(<0.001)		(＜0.001)		(＜0.001)		(＜0.001)		(＜0.001)		(＜0.001)	
Vaccine effectiveness (30%)												
50%	0.687***	0.447	0.985***	0.280	0.770***	0.413	0.677***	0.773***	0.985***	0.280	0.798***	0.136 (0.374)
	(0.105)	(0.300)	(0.107)	(0.382)	(0.101)	(0.296)	(0.109)	(0.191)	(0.107)	(0.382)	(0.103)	
80%	2.451***	1.875***	2.501***	1.671***	2.339***	1.644***	2. 225***	1.645***	2.501***	1.671***	2.670***	1.937***
	(0.197)	(0.198)	(0.194)	(0.187)	(0.182)	(0.181)	(0.176)	(0.179)	(0.194)	(0.187)	(0.204)	(0.193)
Source of recommendation (relatives)												
Physician	0.662***	0.389	0.505***	0.828***	0.472***	0.507**	0.562***	0.409	0.505***	0.828***	0.592***	0.524**
	(0.106)	(0.291)	(0.103)	(0.178)	(0.09)	(0.230)	(0.102)	(0.263)	(0.103)	(0.178)	(0.103)	(0.228)
School	0.244**	0.057	0.230**	0.073	0.281***	0.015	0.326***	0.036	0.230**	0.073	0.231**	0.076
	(0.102)	(0.340)	(0.093)	(0.397)	(0.096)	(0.567)	(0.101)	(0.358)	(0.093)	(0.397)	(0.100)	(0.892)
Duration of vaccination protection (6 months)												
12 months	0.489***	0.464**	0.161*	0.025**	0.299***	0.674***	0.384***	0.660***	0.161*	0.025**	0.419***	0.623***
	(0.093)	(0.237)	(0.084)	(0.302)	(0.091)	(0.193)	(0.093)	(0.182)	(0.084)	(0.302)	(0.095)	(0.215)
Vaccine safety (30%)												
0%	1.881***	1.128***	1.671***	0.887***	1.707***	1.301***	1.712***	1.320***	1.671***	0.887***	1.947***	1.163***
	(0.165)	(0.169)	(0.145)	(0.176)	(0.154)	(0.168)	(0.156)	(0.165)	(0.145)	(0.176)	(0.161)	(0.169)
15%	0.708***	0.200	0.661***	0.110	0.674***	0.015	0.683***	0.052	0.661***	0.110	0.736***	0.077
	(0.107)	(0.462)	(0.100)	(0.489)	(0.100)	(0.249)	(0.105)	(0.341)	(0.100)	(0.489)	(0.105)	(0.425)

Mode of administration (intramuscular injection)												
Nasal spray	-0.058	0.547***	0.119	0.438*	0.164*	0.207	0.075	0.500**	0.119	0.438*	0.045	0.448*
	(0.092)	(0.204)	(0.091)	(0.204)	(0.086)	(0.508)	(0.090)	(0.219)	(0.091)	(0.204)	(0.092)	(0.264)
ASC	-0.083	2.747***	0.637**	2.858***	0.888***	3.324***	0.177	2.637***	0.637**	2.858***	0.574*	3.496***
	(0.264)	(0.246)	(0.279)	(0.265)	(0.322)	(0.307)	(0.254)	(0.245)	(0.279)	(0.265)	(0.324)	(0.317)
AIC	3673.997		3578.563		3712.582		3765.025		3578.563		3619.866	
BIC	3804.465		3718.46		3846.76		3895.396		3718.46		3751.137	
No. of respondents, (n)	394		403		409		392		403		411	
No. of observations, (n)	7092		7254		7362		7056		7254		7398	
Log-likelihood	-1817.9984		-1774.7816		-1837.2911		-1863.5125		-1774.7816		-1790.9332	
Likelihood ratio chi2	424.49		427.31		514.24		387.11		427.31		557.61	

*p<0.1, **p<0.05, ***p<0.01; SD: standard deviation; SE: standard error; East: Guangdong and Liaoning; Central: Hubei and Jiangxi; West: Shaanxi and Yunnan; High: Shaanxi and Liaoning; Middle: Hubei and Jiangxi; Low: Guangdong and Yunnan; AIC: Akaike Information Criterion; BIC: Bayesian Information Criterion; ASC: Alternative Specific Constant.

Supplementary Figure S1 Locations of parents from six province in China

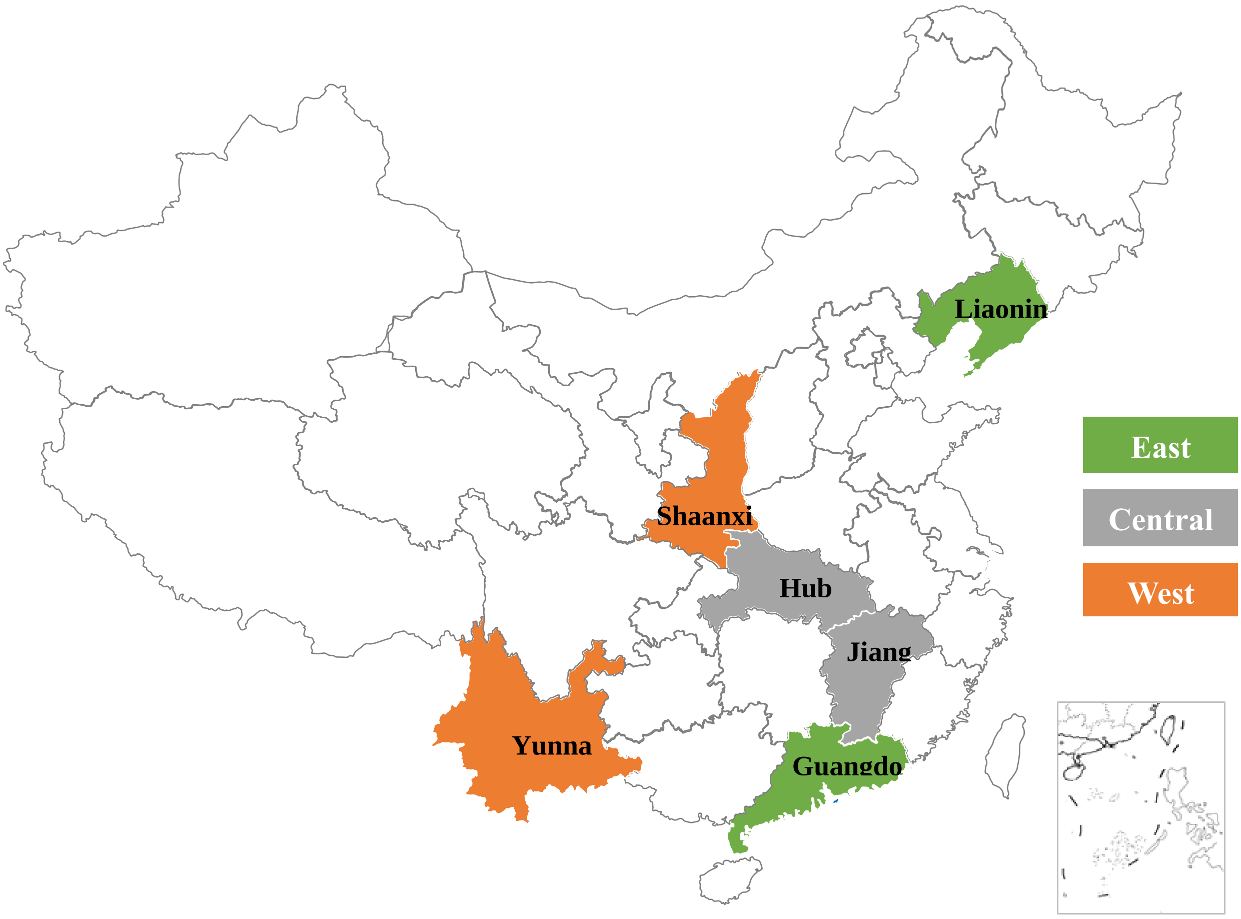

Supplement: Supplementary file 1 [file ijerph-19-02145-s001.zip › ijerph-1521258-supplementary.pdf]
